# Clinical Characteristics and Early Pain Outcomes After Microvascular Decompression for Trigeminal Neuralgia: A Single-Center Retrospective Cohort Study

**DOI:** 10.3390/brainsci16080891

**Published:** 2026-08-20

**Authors:** Yasemin Adalı, Ümit Akın Dere

**Affiliations:** 1 Centre for Public Health, School of Medicine, Dentistry and Biomedical Sciences, Queen’s University Belfast, Belfast BT12 6BJ, UK; 2 Epidemiology Division, Department of Public Health, Medical Faculty, Pamukkale University, Denizli 20160, Türkiye; 3 Department of Neurosurgery, Medical Faculty, Pamukkale University, Denizli 20160, Türkiye

**Keywords:** trigeminal neuralgia, microvascular decompression surgery, pain measurement, retrospective studies, postoperative outcomes

## Abstract

Background: Trigeminal neuralgia is a chronic neuropathic pain disorder characterized by severe recurrent facial pain and substantial impairment in quality of life. Although microvascular decompression (MVD) is an established surgical treatment for medically refractory cases, routine clinical data integrating clinical characteristics, previous interventional treatment history, and early postoperative outcomes remain limited. This study aimed to describe the clinical profile and early postoperative outcomes of patients undergoing MVD for trigeminal neuralgia in a single-center retrospective cohort. Methods: This single-center, single-surgeon retrospective cohort study included 62 adult patients who underwent MVD at Pamukkale University Hospitals between 4 September 2019 and 9 July 2026. Demographic and clinical characteristics, previous interventional procedures, trigeminal nerve division involvement, vascular compression status, surgical opening approach, postoperative Barrow Neurological Institute (BNI) pain intensity scores, visual analog scale (VAS) pain scores, wound-related complications, and exploratory perioperative inflammatory markers were retrospectively collected. Favorable postoperative outcome was defined as BNI Grade I–II. Results: The mean age was 51.9 ± 12.8 years, 66.1% were female, and vascular compression was identified in 87.1%. A previous interventional procedure was documented in 35.5%. Favorable BNI outcomes occurred in 74.2% (95% CI, 62.1–83.4%), and complete pain relief (VAS = 0) in 88.7% (95% CI, 78.5–94.4%). Wound-related complications occurred in 9.7%. No evaluated baseline characteristic was significantly associated with either pain outcome. Paired analyses showed no significant perioperative change in CRP (*p* = 0.353), whereas NLR increased significantly (*p* = 0.001). Conclusions: MVD was associated with favorable early postoperative pain outcomes in most patients. However, the retrospective design, modest sample size, and symptom-driven longer-term follow-up limit conclusions regarding prognostic determinants and durability of pain relief.

## 1. Introduction

Trigeminal neuralgia is a chronic neuropathic pain disorder characterized by sudden, severe, recurrent episodes of facial pain that can substantially impair daily functioning and quality of life [1]. Although trigeminal neuralgia is relatively uncommon, it represents a clinically important craniofacial pain condition because of its intensity, diagnostic complexity, frequent need for long-term treatment, and potential requirement for surgical management in medically refractory cases [1,2,3,4].

Medical therapy is generally considered the first-line treatment for trigeminal neuralgia, while surgical treatment is considered in patients with medically refractory symptoms or poor tolerance to medication [1,2]. Carbamazepine and oxcarbazepine are recommended as first-line pharmacological treatments for trigeminal neuralgia, while lamotrigine, gabapentin, pregabalin, baclofen, phenytoin, and botulinum toxin type A may be considered as alternative or add-on therapies. Intravenous fosphenytoin or lidocaine may also be used for severe acute exacerbations requiring hospital treatment [5]. For patients with persistent symptoms despite pharmacological treatment, microvascular decompression (MVD) is one of the principal surgical options because it directly addresses neurovascular conflict at the trigeminal nerve root entry zone and may provide durable pain relief in appropriately selected patients [2,6,7,8]. However, postoperative outcomes after MVD are not uniform, and pain relief, recurrence, complications, and the need for further intervention may vary according to patient characteristics, disease features, neurovascular compression, and perioperative factors [6,7,8,9,10].

The pathophysiology of trigeminal neuralgia is complex and not yet fully resolved, although neurovascular compression and focal demyelination are among the most widely accepted mechanisms [1,3]. At the same time, the clinical course of surgically treated patients is not uniform, and postoperative outcomes may vary according to patient characteristics, disease features, and perioperative findings [2,3,10]. This makes descriptive cohort studies valuable not only for documenting outcomes, but also for improving the overall clinical characterization of surgically treated populations [2,10].

Many MVD studies have focused on long-term pain freedom, recurrence, or surgical technique. Additional descriptive cohorts that integrate demographic and clinical characteristics with postoperative pain outcomes, complication patterns, postoperative management, and perioperative clinical variables may provide complementary clinical information within a consistent surgical setting [6,7,8,9,10,11]. Such studies can place postoperative recovery within the broader clinical profile of surgically treated trigeminal neuralgia patients. Accordingly, retrospective surgical cohorts that integrate patient characteristics, operative findings, postoperative pain outcomes, and perioperative clinical variables remain useful [7,10,11].

Systemic inflammatory markers such as C-reactive protein (CRP) and the neutrophil-to-lymphocyte ratio (NLR) are routinely available perioperative laboratory parameters and have been explored in neurological and neurosurgical conditions [12,13,14]. However, their relevance in trigeminal neuralgia and after MVD remains uncertain [12,13]. Therefore, in the present study, CRP and NLR were evaluated only as exploratory perioperative markers rather than as established biomarkers of trigeminal neuralgia severity or surgical success.

The present study provides an integrated descriptive evaluation of demographic and clinical characteristics, previous interventional treatment history, neurovascular compression findings, postoperative BNI- and VAS-based pain outcomes, wound-related complications, postoperative management, and exploratory perioperative inflammatory markers within a consecutive single-center, single-surgeon MVD cohort. Rather than providing a large long-term efficacy estimate, the study contributes a contemporary, clinically integrated cohort from western Türkiye and complements larger MVD series that have primarily focused on long-term pain freedom, recurrence, or selected prognostic factors. Because of the limited sample size and retrospective design, the study was framed as an exploratory descriptive cohort rather than a definitive prognostic study. Accordingly, this study aimed to describe the clinical characteristics of patients who underwent MVD for trigeminal neuralgia and to evaluate early postoperative outcomes in a single-center, single-surgeon retrospective cohort.

## 2. Materials and Methods

### 2.1. Study Design, Setting, and Ethical Approval

This study was designed as a single-center, single-surgeon, retrospective cohort study conducted at Pamukkale University Hospitals, Department of Neurosurgery, Denizli, Türkiye. The study protocol was approved by the Pamukkale University Non-Interventional Clinical Research Ethics Committee, Pamukkale University, Denizli, Türkiye, before data analysis was performed (protocol code E-60116787-020-828645; approval date: 12 February 2026). The study was conducted in accordance with the Declaration of Helsinki. Medical records of adult patients who underwent microvascular decompression (MVD) for trigeminal neuralgia between 4 September 2019 and 9 July 2026 were retrospectively reviewed. The study period was extended to include all additional eligible consecutive patients who underwent MVD up to 9 July 2026. Following this extension, the dataset was updated and all analyses were repeated using the final cohort of 62 patients. All surgical procedures were performed by the same neurosurgeon. The study was conducted as a non-interventional archive-based clinical investigation, and no additional clinical examination, laboratory test, or intervention was performed for research purposes. All data were anonymized before analysis.

### 2.2. Study Population

Patients were eligible for inclusion if they met all the following criteria: (1) diagnosis of trigeminal neuralgia, (2) treatment with MVD at the study center during the study period, and (3) availability of sufficient medical records for analysis. This study included patients with trigeminal neuralgia who underwent MVD and did not include other facial pain syndromes. The term facial pain was used only to describe the clinical manifestation of trigeminal neuralgia. The clinical diagnosis of trigeminal neuralgia was based on the characteristic clinical presentation documented in the medical records and was consistent with the diagnostic framework of the International Classification of Headache Disorders, 3rd edition (ICHD-3), including recurrent unilateral paroxysmal facial pain within the distribution of one or more divisions of the trigeminal nerve, typically severe and shock-like or stabbing in character and triggered by innocuous stimuli. Potential secondary causes were assessed from the available clinical history, neurological examination, neuroimaging, and operative records.

Patients with an identifiable underlying condition capable of explaining the facial pain, including multiple sclerosis, intracranial tumor, postherpetic trigeminal neuropathy, or another secondary facial pain disorder, were excluded when identifiable from the available records. Although the clinical diagnosis of trigeminal neuralgia was consistent with the ICHD-3 framework, the retrospective records did not consistently contain the detailed imaging and/or intraoperative morphological information required to reliably distinguish classical from idiopathic trigeminal neuralgia. Therefore, patients were not retrospectively subclassified as classical or idiopathic TN. Neurovascular compression was instead recorded and analysed separately as an operative finding. Patients with missing key clinical or follow-up data were also excluded because postoperative outcome classification could not be reliably assigned. After application of the eligibility criteria and exclusion of repeated or incomplete records, the final analytic cohort included 62 patients.

All eligible consecutive patients identified within the study period were included in the analysis; therefore, no formal a priori sample size or power calculation was performed. The study should be interpreted as an exploratory retrospective cohort rather than a hypothesis-confirming prognostic study.

### 2.3. Surgical Technique

Microvascular decompression was performed in patients with medically refractory trigeminal neuralgia or in patients who could not tolerate medical treatment. Surgical indication was based on clinical diagnosis, symptom severity, response to medical therapy, preoperative radiological evaluation, and neurosurgical assessment. Routine preoperative imaging included brain MRI, MR angiography, and thin-slice FIESTA sequences to evaluate the trigeminal nerve and possible neurovascular conflict. All procedures were performed by the same neurosurgeon using a standard retrosigmoid approach. After positioning and retrosigmoid craniotomy, the cerebellopontine angle was exposed microsurgically, and the trigeminal nerve root entry zone was inspected. When neurovascular compression was identified, the offending vessel was carefully separated from the trigeminal nerve root entry zone. When neurovascular compression was identified, Teflon felt was interposed between the offending vessel and the trigeminal nerve to maintain decompression.

Intraoperative electrophysiological monitoring was not routinely used in this cohort. In this series of patients undergoing MVD for trigeminal neuralgia, operative decision-making was based primarily on microsurgical exposure of the trigeminal nerve root entry zone, identification of neurovascular compression, and adequate decompression. Detailed grading of neurovascular compression severity, including contact, displacement, indentation, or nerve-root distortion, was not consistently documented and could not be reliably reconstructed from the retrospective operative records. In patients without identifiable neurovascular compression, arachnoid adhesions surrounding the trigeminal nerve that were considered to contribute to neural tension were carefully released, and adequate relaxation of the trigeminal nerve was confirmed intraoperatively.

### 2.4. Data Collection and Variables

Patients were identified through the hospital information management system, operative records, and departmental archives. Data were retrospectively extracted from patient files, discharge summaries, operative notes, and follow-up records. The recorded variables included demographic characteristics such as age and sex, clinical characteristics such as side of pain, trigeminal nerve division involvement, duration of symptoms, and comorbidity status, perioperative findings such as the presence of vascular compression, surgical opening approach, and documented previous interventional procedures before the index MVD. Previous MVD was recorded separately to distinguish patients undergoing primary MVD from those undergoing repeat or revision MVD. Postoperative outcomes included Barrow Neurological Institute (BNI) pain intensity scores, visual analog scale (VAS) pain scores, wound-related complications, postoperative management for wound-related complications, post-index reintervention when documented, and follow-up data.

Preoperative and postoperative inflammatory markers, including C-reactive protein (CRP) and neutrophil to lymphocyte ratio (NLR), were collected when available from routine clinical records. CRP and NLR were included as exploratory variables because they were routinely available from perioperative laboratory records. These markers were not considered established biomarkers of trigeminal neuralgia severity or MVD success, and their analysis was intended to describe perioperative systemic inflammatory changes rather than to provide prognostic conclusions. Preoperative CRP and NLR values were obtained from blood samples collected immediately before surgery, whereas postoperative CRP and NLR values were obtained from blood samples collected 48 h after surgery.

### 2.5. Outcomes and Follow-Up

Descriptive epidemiological and clinical characteristics were evaluated to characterize the study cohort, including age, sex, side of pain, trigeminal nerve division involvement, symptom duration, comorbidity status, and vascular compression status. The primary outcome was favorable postoperative pain control. For the main analysis, a favorable postoperative outcome was defined as a BNI pain intensity score of Grade I or II, whereas Grades III and IV were classified as unfavorable [15]. BNI Grades I–II were considered favorable because they represent complete pain relief or only occasional pain not requiring medication, which is commonly interpreted as successful postoperative pain control in surgical series of trigeminal neuralgia [16]. A secondary pain-related outcome was complete pain relief, defined as a postoperative VAS score of 0. Pain intensity was assessed using the VAS on a 0–10 scale, with 0 representing no pain and 10 representing the worst imaginable pain [17]. BNI and VAS were used as complementary outcome measures. The BNI pain intensity scale was selected as the primary outcome because it captures clinically meaningful postoperative pain control and medication status, whereas VAS was used as a secondary measure of pain intensity, with VAS = 0 indicating complete pain relief. Postoperative BNI grades were extracted from the documented clinical follow-up records, while detailed postoperative medication changes were not consistently available for separate analysis.

Postoperative BNI pain intensity scores and VAS scores were assessed at the routine 1-month postoperative neurosurgical follow-up and were extracted from the same documented clinical assessment. Exact postoperative day-level timing was not consistently available in the retrospective records. Patients with persistent, recurrent, or worsening pain were subsequently re-evaluated as clinically indicated. Because follow-up beyond the routine early postoperative period was symptom-driven and was not standardized across all patients, the present study focused on early postoperative pain outcomes rather than long-term durability of pain relief. Secondary outcomes also included wound-related complications, postoperative management for wound-related complications, and perioperative changes in CRP and NLR values. Wound-related complications were defined as documented CSF leakage or CSF-related wound complications, wound discharge, and pseudomeningocele occurring during the available postoperative follow-up. The recorded timing of these events ranged from 2 weeks to 1 year after surgery. No wound infection was documented.

### 2.6. Statistical Analysis

Statistical analyses were performed using Stata IC version 16.1 (StataCorp LLC, College Station, TX, USA). Continuous variables were summarized as mean ± standard deviation or median (interquartile range [IQR]) depending on the distribution, and categorical variables were presented as number and percentage. Categorical variables were compared between outcome groups using Fisher’s exact test, where appropriate, and continuous variables were compared using the Mann–Whitney U test. Preoperative and postoperative CRP and NLR values were compared using the Wilcoxon signed-rank test among patients with available paired measurements. For the two main postoperative pain outcome proportions, 95% confidence intervals were calculated using the Wilson method. For categorical exploratory comparisons, absolute differences in outcome proportions were additionally reported with 95% confidence intervals calculated using the Newcombe method based on Wilson score intervals. Because the outcome-group comparisons were exploratory and the number of unfavorable outcomes was limited, emphasis was placed on exact two-sided *p* values and clinically interpretable group estimates rather than on effect-size classification. Because of the small number of unfavorable postoperative outcomes, multivariable regression analysis was not performed to avoid overfitting and unstable estimates. Therefore, analyses of factors associated with postoperative outcomes should be interpreted as exploratory. Sensitivity analyses were performed by excluding: (1) patients with isolated V1 involvement, (2) patients without vascular compression, and (3) patients with comorbidities. These sensitivity analyses involved recalculation of the principal outcome rates rather than repeated hypothesis testing. All tests were two-sided, and *p* values < 0.05 were considered statistically significant.

## 3. Results

**Figure 1:**
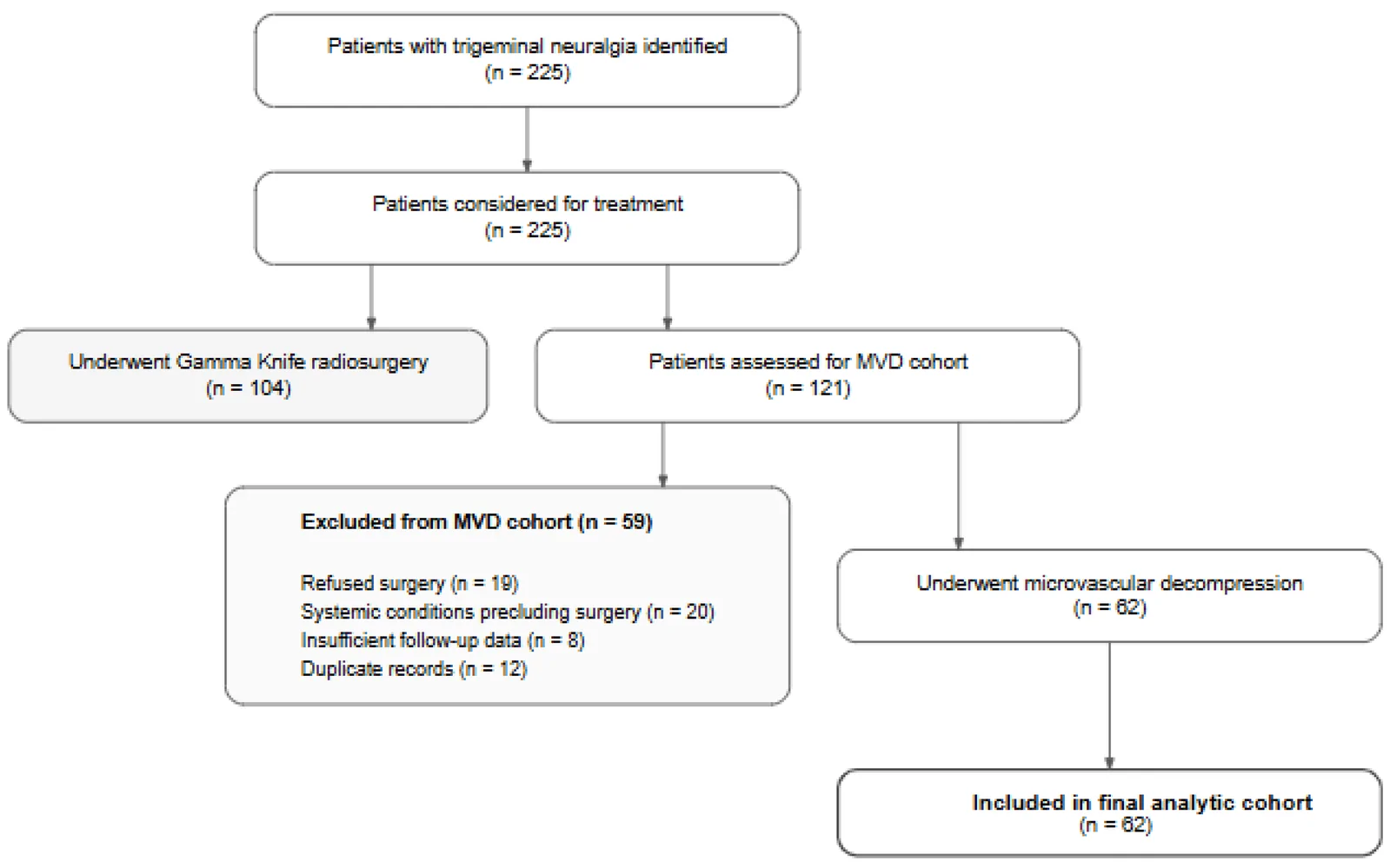
Flow diagram of patient identification, treatment pathway, and inclusion in the final microvascular decompression cohort.

A total of 62 patients with trigeminal neuralgia who underwent MVD between 4 September 2019 and 9 July 2026 were included in the final analysis. The patient identification and selection process is shown in Figure 1.

The mean age was 51.9 ± 12.8 years, and the median age was 54 years (IQR, 40–62). Females comprised 66.1% of the cohort (*n* = 41), while males accounted for 33.9% (*n* = 21). Right-sided disease was slightly more common than left-sided disease (53.2% vs. 46.8%). The mean symptom duration was 7.9 ± 6.5 years, with a median duration of 5.5 years (IQR, 3–10). Comorbidities were present in 37.1% of patients, and vascular compression was identified in 87.1% of cases (Table 1).

A documented previous interventional procedure before the index MVD was present in 22 patients (35.5%), most commonly radiofrequency procedures, Gamma Knife radiosurgery, or previous MVD. The surgical opening approach was retrosigmoid craniotomy in 53 patients (85.5%) and craniectomy/cranioplasty variants in 9 patients (14.5%). Regarding trigeminal nerve division involvement, V2/3 involvement was the most frequent pattern (24.2%), followed by combined V1/2/3 involvement (22.6%) and isolated V2 involvement (19.4%). Isolated V1 involvement was uncommon (3.2%). The distribution of trigeminal division involvement is shown in Table 1. Preoperative medication records showed that carbamazepine was the most documented treatment (59/62, 95.2%), followed by gabapentin (25/62, 40.3%), pregabalin (22/62, 35.5%), baclofen (8/62, 12.9%), and duloxetine (8/62, 12.9%). Oxcarbazepine was documented in two patients (3.2%). Medication categories were not mutually exclusive because many patients had received combination pharmacotherapy (Table 1).

Early postoperative pain outcomes and documented wound-related complications are summarized in Table 2. According to the postoperative BNI classification, 44 patients (71.0%) had Grade I outcomes, 2 (3.2%) had Grade II, 13 (21.0%) had Grade III, and 3 (4.8%) had Grade IV. Overall, favorable postoperative BNI outcomes (Grade I-II) were observed in 46 patients (74.2%; 95% CI, 62.1–83.4%), whereas 16 patients (25.8%) had unfavorable outcomes (Grade III-IV). Complete pain relief, defined as a postoperative VAS score of 0, was achieved in 55 patients (88.7%; 95% CI, 78.5–94.4%). BNI and VAS were interpreted as complementary rather than interchangeable outcomes because VAS reflects pain intensity at the documented assessment, whereas BNI also incorporates medication status and clinically meaningful pain control. Cross-tabulation of postoperative BNI and VAS outcomes showed that all 46 patients classified as BNI Grades I–II had a VAS score of 0. Among the 16 patients classified as BNI Grades III–IV, nine had a VAS score of 0 and seven had residual pain (VAS > 0), illustrating the complementary nature of the two outcome measures.

Wound-related complications were observed in six patients (9.7%) and included cerebrospinal fluid leakage or CSF-related wound complications in two patients, wound discharge in two patients, and pseudomeningocele in two patients. Postoperative management included surgical revision in two patients, dressing or wound care in one patient, and clinical follow-up without further intervention in three patients. One patient underwent post-index Gamma Knife radiosurgery 2 years after the index MVD during follow-up; no repeat MVD after the index operation was documented in the available records.

Preoperative and postoperative inflammatory markers are presented in Table 3. Among 59 patients with complete paired CRP measurements, median CRP was 2.55 (IQR, 1.39–8.94) preoperatively and 2.59 (IQR, 1.12–5.30) postoperatively; this change was not statistically significant (*p* = 0.353). Among 61 patients with complete paired NLR measurements, median NLR increased from 1.91 (IQR, 1.56–2.34) preoperatively to 2.73 (IQR, 1.68–4.79) postoperatively (*p* = 0.001). These findings were interpreted descriptively because CRP and NLR were exploratory perioperative markers rather than validated indicators of trigeminal neuralgia severity or surgical success.

Factors associated with favorable postoperative BNI outcome are shown in Table 4. No statistically significant associations were identified between favorable BNI outcome and sex (*p* = 0.220), disease side (*p* = 0.780), vascular compression status (*p* = 0.414), comorbidity status (*p* = 0.242), previous interventional procedure before the index MVD (*p* = 0.546), age (*p* = 0.521), or symptom duration (*p* = 0.425). Absolute differences in favorable outcome proportions with 95% confidence intervals are also presented in Table 4 to provide estimates of effect size and precision. These findings should be interpreted cautiously given the modest sample size and limited number of unfavorable outcomes.

Similarly, none of the evaluated baseline characteristics showed a statistically significant association with complete pain relief, defined as postoperative VAS = 0. No significant differences were observed according to sex (*p* = 1.000), disease side (*p* = 0.237), vascular compression status (*p* = 0.580), comorbidity status (*p* = 1.000), previous interventional procedure before the index MVD (*p* = 0.086), age (*p* = 0.559), or symptom duration (*p* = 0.858) (Table 5). Complete pain relief was observed in 95.0% of patients without a documented previous interventional procedure and in 77.3% of those with a previous intervention, corresponding to an absolute difference of 17.7 percentage points (95% CI, 0.7–38.8). Although the prespecified two-sided Fisher exact test was not statistically significant (*p* = 0.086), the magnitude and precision of this difference are reported descriptively given the exploratory nature and limited sample size of the study.

Among the six patients who had undergone a previous MVD, favorable BNI outcome was observed in 3 patients (50.0%) and complete pain relief was achieved in 4 patients (66.7%). In comparison, among the 56 patients undergoing primary MVD, favorable BNI outcome was observed in 43 patients (76.8%) and complete pain relief in 51 patients (91.1%). Given the small number of repeat or revision MVD cases, these subgroup findings were interpreted descriptively.

Sensitivity analyses were performed to assess the robustness of the primary outcome findings. Excluding the two patients with isolated V1 involvement, favorable BNI outcome was observed in 45 of 60 patients (75.0%), and complete pain relief was achieved in 53 of 60 patients (88.3%). When the eight patients without vascular compression were excluded, favorable BNI outcome was observed in 41 of 54 patients (75.9%), and complete pain relief was achieved in 47 of 54 patients (87.0%). Similarly, exclusion of the 23 patients with comorbidities yielded a favorable BNI outcome in 31 of 39 patients (79.5%) and complete pain relief in 35 of 39 patients (89.7%). In a further sensitivity analysis excluding the six patients with previous MVD, favorable BNI outcome was observed in 43 of 56 patients (76.8%), while complete pain relief was achieved in 51 of 56 patients (91.1%). Finally, when all 22 patients with any previous interventional procedure were excluded, favorable BNI outcome was observed in 31 of 40 patients (77.5%), and complete pain relief was achieved in 38 of 40 patients (95.0%). Overall, these sensitivity analyses yielded findings that were broadly consistent with the main analysis.

## 4. Discussion

In this single-center, single-surgeon retrospective cohort, microvascular decompression (MVD) was associated with favorable early postoperative pain control in most patients with trigeminal neuralgia. Favorable postoperative BNI outcome was observed in 74.2% of patients, and complete pain relief was achieved in 88.7%. These findings should be interpreted as descriptive early postoperative outcomes rather than definitive evidence regarding patient-level determinants or long-term durability of pain relief because postoperative pain outcomes were primarily based on early follow-up and longer-term follow-up was symptom-driven rather than uniformly standardized [1,6,8,18,19].

Our pain outcome findings are broadly consistent with the long-term literature supporting MVD as an effective surgical treatment for trigeminal neuralgia. In the landmark study by Barker et al., 70% of patients remained pain-free without medication at 10 years, and the annual recurrence rate after 10 years was reported to be less than 1% [8]. Similarly, Sarsam et al. reported complete pain relief without medication in 71% of patients at 10 years and an overall satisfaction rate of 84% [6]. More recently, Worm et al. showed in a prospective observational cohort that MVD provided superior long-term pain relief and higher rates of medication-free pain freedom than medical management alone, further supporting the role of surgery in appropriately selected patients [18]. However, direct comparison with these long-term studies is limited because BNI and VAS outcomes in the present cohort were based on the routine 1-month postoperative assessment, whereas subsequent follow-up was mainly symptom-driven. Therefore, the high rate of complete pain relief observed in our study should be interpreted as reflecting early postoperative pain control rather than long-term durability of pain relief.

The demographic and clinical profile of our cohort provides additional clinical information on surgically treated trigeminal neuralgia patients. The median age was 54 years, females were more common than males, and right-sided disease was slightly more frequent than left-sided disease. Vascular compression was identified in 87.1% of cases, supporting the role of neurovascular conflict in surgically selected trigeminal neuralgia patients [1,20]. V2/3 involvement was the most frequent trigeminal division pattern, followed by combined V1/2/3 and isolated V2 involvement. More than one-third of patients had documented previous interventional procedures before the index MVD, including radiofrequency procedures, Gamma Knife radiosurgery, previous MVD, nerve or foramen blocks, SPG block, cryotherapy, or balloon compression. This treatment-exposed subgroup may represent a clinically more complex population; however, the present study was not powered to establish the prognostic effect of previous procedures.

No statistically significant associations were identified between favorable postoperative BNI outcome and the evaluated baseline variables, including sex, disease side, vascular compression, comorbidity status, previous intervention, age, and symptom duration. Similarly, no evaluated baseline variable was significantly associated with complete pain relief. Complete pain relief was numerically lower in patients with a previous intervention (77.3%) than in those without one (95.0%), but the two-sided Fisher exact test did not show statistical significance (*p* = 0.086). These findings should be interpreted cautiously because of the modest sample size and small number of patients with residual pain or unfavorable BNI outcomes.

Previous studies have identified several factors associated with postoperative outcomes after MVD. Huang et al. reported that atypical trigeminal neuralgia and severe venous compression were associated with poorer drug-free outcomes after MVD [20]. Menna et al. found that the presence of an offending artery with nerve root distortion or indentation was an independent prognostic factor for pain-free survival, while clinical outcomes were otherwise comparable between younger and elderly patients [11]. Barker et al. also identified female sex, longer symptom duration, venous compression, and lack of immediate postoperative cessation of pain as predictors of eventual recurrence [8]. In contrast, no clear baseline predictors of favorable postoperative pain control were identified in the present cohort. This should be interpreted cautiously in light of the exploratory design, limited number of unfavorable outcomes, and differences in cohort characteristics, follow-up duration, and outcome definitions across studies. Vascular compression was consistently recorded as present or absent; however, arterial, venous, or mixed compression subtype was not uniformly documented in the retrospective operative records and therefore could not be analysed separately.

The observed wound-related complication rate was 9.7%. Wound-related complications included cerebrospinal fluid leakage or CSF-related wound complications, wound discharge, and pseudomeningocele, and these events were reported separately to improve clinical interpretability. Postoperative management included surgical revision in two patients, dressing or wound care in one patient, and clinical follow-up without further intervention in three patients. One patient subsequently underwent Gamma Knife radiosurgery 2 years after the index MVD, and no repeat MVD after the index operation was documented. These findings should be interpreted in the context of the small cohort size and the broad retrospective definition of wound-related complications. Larger MVD series have generally reported low rates of major complications, although reported complication rates vary according to outcome definitions, follow-up duration, surgical technique, and whether minor wound-related events are included. Therefore, our complication findings are best interpreted descriptively rather than as precise estimates of surgical risk. Nevertheless, reporting wound-related complications together with pain outcomes is clinically important because the value of MVD should not be assessed solely in terms of pain relief [6,8,18].

CRP did not show a statistically significant paired preoperative-to-postoperative change, whereas NLR increased significantly 48 h after surgery. These findings should be interpreted cautiously. Previous studies have suggested that inflammatory mechanisms may be involved in trigeminal neuralgia, and inflammatory biomarkers have been investigated in both cerebrospinal fluid and peripheral blood [21,22,23]. However, CRP and NLR are not established biomarkers of trigeminal neuralgia severity or MVD success. The postoperative NLR increase may reflect nonspecific perioperative physiological and stress responses, timing of blood sampling, medications, and comorbid conditions rather than a disease-specific mechanism. Accordingly, CRP and NLR should remain exploratory perioperative variables in the present study.

The burden of trigeminal neuralgia extends beyond pain intensity alone. Bauer et al. reported significant improvements in depression and perceived stress after MVD, together with improvement in BNI pain scores, suggesting that successful pain control may also contribute to better psychological well-being [12]. Although quality of life and psychiatric symptoms were not assessed in the present study, these findings provide additional clinical context for the importance of postoperative pain control [12,18,24].

This study has several strengths. It reflects a consecutive surgical cohort from a single center, and all procedures were performed by the same neurosurgeon, reducing inter-operator variability in surgical technique and perioperative decision-making. In addition, the analysis integrated demographic and clinical characteristics with postoperative BNI and VAS-based pain outcomes, wound-related complications, postoperative management, perioperative inflammatory markers, and sensitivity analyses within the same cohort. This integrated approach allowed demographic, clinical, operative, postoperative, and perioperative variables to be evaluated within the same cohort [6,11,20].

Several limitations should also be acknowledged. First, the retrospective design limited standardization of variable collection and made the analysis dependent on the completeness and quality of archived medical records. Second, the sample size was relatively small, and the number of unfavorable outcomes was limited; therefore, multivariable regression analysis was not performed to avoid overfitting and unstable estimates. Third, postoperative BNI and VAS scores were based on the routine 1-month postoperative neurosurgical follow-up assessment, while longer-term follow-up was symptom-driven and not uniform across all patients. Exact postoperative day-level timing of the 1-month assessment was not consistently available in the retrospective records. Fourth, selection bias is possible because the cohort included only patients who underwent MVD and excluded patients with missing key clinical or follow-up data. Fifth, detailed classification of vascular compression as arterial, venous, or mixed was not consistently available in the retrospective operative records; therefore, vascular compression was analysed only as present or absent. Sixth, intraoperative electrophysiological monitoring was not routinely used, which may limit comparability with some contemporary MVD series. Seventh, atypical trigeminal neuralgia subtypes, standardized quality-of-life or psychiatric outcomes, and detailed postoperative medication discontinuation or reduction were not consistently documented and therefore could not be analysed. In addition, other clinically relevant complications of MVD, including hearing loss, cranial nerve deficits, sensory disturbances, infection, hematoma, cerebrovascular complications, and mortality, could not be systematically assessed because these outcomes were not consistently documented in the retrospective records. Therefore, the reported 9.7% rate should be interpreted specifically as the rate of documented wound-related complications rather than as an overall surgical complication rate. Finally, CRP and NLR were exploratory perioperative markers and should not be interpreted as validated biomarkers of trigeminal neuralgia severity, postoperative pain relief, or surgical success.

## 5. Conclusions

Overall, MVD was associated with favorable early postoperative pain outcomes in most patients with trigeminal neuralgia in this single-center, single-surgeon retrospective cohort. No clear baseline predictors of favorable postoperative outcome or complete pain relief were identified. These findings should be interpreted cautiously in light of the retrospective design, modest sample size, early postoperative outcome assessment, and potential selection bias. Larger prospective multicenter studies with standardized follow-up, detailed imaging and operative characterization of neurovascular compression, and longer-term assessment of recurrence are needed to better clarify predictors of surgical success and durability of pain relief.

## Figures and Tables

**Table 1:** Baseline demographic and clinical characteristics of patients with trigeminal neuralgia.

Variable	Value
Number of patients	62
Age, mean ± SD	51.9 ± 12.8
Age, median (IQR)	54 (40–62)
Female, n (%)	41 (66.1)
Male, n (%)	21 (33.9)
Right-sided disease, n (%)	33 (53.2)
Left-sided disease, n (%)	29 (46.8)
Symptom duration, mean ± SD, years	7.9 ± 6.5
Symptom duration, median (IQR), years	5.5 (3–10)
Comorbidity present, n (%)	23 (37.1)
No comorbidity, n (%)	39 (62.9)
Vascular compression present, n (%)	54 (87.1)
Vascular compression absent, n (%)	8 (12.9)
Trigeminal nerve division involvement, n (%)	
V1	2 (3.2)
V1/2	8 (12.9)
V1/2/3	14 (22.6)
V2	12 (19.4)
V2/3	15 (24.2)
V3	11 (17.7)
Previous interventional procedures before index MVD, n (%)	
None	40 (64.5)
One procedure	17 (27.4)
Two or more procedures	5 (8.1)
Radiofrequency procedure	12 (19.4)
Gamma Knife radiosurgery	7 (11.3)
Previous MVD	6 (9.7)
Nerve block	2 (3.2)
SPG block	1 (1.6)
Cryotherapy	1 (1.6)
Foramen block	1 (1.6)
Balloon compression	1 (1.6)
Post-index Gamma Knife radiosurgery during follow-up, n (%)	1 (1.6)
Preoperative medication use, n (%)	
Carbamazepine	59 (95.2)
Gabapentin	25 (40.3)
Pregabalin	22 (35.5)
Baclofen	8 (12.9)
Duloxetine	8 (12.9)
Oxcarbazepine	2 (3.2)
No documented medication	1 (1.6)
Surgical opening approach, n (%)	
Retrosigmoid craniotomy	53 (85.5)
Retrosigmoid craniectomy/cranioplasty variants	9 (14.5)

Previous interventional procedure categories in Table 1 are not mutually exclusive because some patients had more than one documented procedure. Post-index Gamma Knife radiosurgery refers to an additional procedure during follow-up and was not counted as a previous interventional procedure before the index MVD.

**Table 2:** Early postoperative pain outcomes and documented wound-related complications.

Variable	Value
Wound-related complication, n (%)	6 (9.7)
Wound-related complication type, n (%)	
CSF leakage/CSF-related wound complication	2 (3.2)
Wound discharge	2 (3.2)
Pseudomeningocele	2 (3.2)
Management of wound-related complications, n (%)	6 (9.7)
Surgical revision	2 (3.2)
Dressing/wound care	1 (1.6)
Clinical follow-up without further intervention	3 (4.8)
Post-index Gamma Knife radiosurgery during follow-up, n (%)	1 (1.6)
Repeat MVD after index operation, n (%)	0 (0.0)
Postoperative BNI grade, n (%)	
Grade I	44 (71.0)
Grade II	2 (3.2)
Grade III	13 (21.0)
Grade IV	3 (4.8)
Favorable BNI outcome (Grade I–II), n (%)	46 (74.2)
Unfavorable BNI outcome (Grade III–IV), n (%)	16 (25.8)
Complete pain relief (postoperative VAS = 0), n/N (%)	55/62 (88.7)
Residual pain (postoperative VAS > 0), n/N (%)	7/62 (11.3)

BNI and VAS should be interpreted as complementary outcomes. VAS = 0 reflects pain intensity at the documented assessment, whereas BNI incorporates pain control and medication status.

**Table 3:** Preoperative and postoperative inflammatory markers.

Variable	Preoperative	Postoperative	*p* Value
CRP, median (IQR)	2.55 (1.39–8.94) [*n* = 59]	2.59 (1.12–5.30) [*n* = 59]	0.353
NLR, median (IQR)	1.91 (1.56–2.34) [*n* = 61]	2.73 (1.68–4.79) [*n* = 61]	0.001

Values are presented as median (IQR). Comparisons were performed using the Wilcoxon signed-rank test among patients with available paired preoperative and postoperative measurements.

**Table 4:** Comparison of baseline characteristics according to postoperative BNI outcome.

Variable	Unfavorable BNI (*n* = 16)	Favorable BNI (*n* = 46)	Absolute Difference (95% CI)	*p* Value
Female, n (%)	13 (81.3)	28 (60.9)	−17.4 (−35.3 to 6.3)	0.220
Male, n (%)	3 (18.8)	18 (39.1)	Reference	
Left-sided disease, n (%)	8 (50.0)	21 (45.7)	−3.3 (−24.8 to 17.8)	0.780
Right-sided disease, n (%)	8 (50.0)	25 (54.3)	Reference	
Vascular compression present, n (%)	13 (81.3)	41 (89.1)	13.4 (−13.6 to 46.7)	0.414
Vascular compression absent, n (%)	3 (18.8)	5 (10.9)	Reference	
Comorbidity present, n (%)	8 (50.0)	15 (32.6)	−14.3 (−36.8 to 7.7)	0.242
No comorbidity, n (%)	8 (50.0)	31 (67.4)	Reference	
Age, median (IQR)	53.5 (44–65)	54 (40–61)	—	0.521
Symptom duration, median (IQR), years	6 (3.5–13)	5.5 (3–9)	—	0.425
Previous interventional procedure before index MVD, n (%)	7 (43.8)	15 (32.6)	−9.3 (−32.5 to 12.2)	0.546
No documented previous interventional procedure before index MVD, n (%)	9 (56.3)	31 (67.4)	Reference	

Categorical variables were compared using two-sided Fisher’s exact test, and continuous variables were compared using the Mann–Whitney U test. Absolute differences represent differences in the proportion of patients with favorable BNI outcome between the listed category and the corresponding reference category and are presented as percentage points with 95% confidence intervals.

**Table 5:** Comparison of baseline characteristics according to complete pain relief status.

Variable	Residual Pain (*n* = 7)	Pain-Free (*n* = 55)	Absolute Difference (95% CI)	*p* Value
Female, n (%)	5 (71.4)	36 (65.5)	−2.7 (−17.7 to 17.9)	1.000
Male, n (%)	2 (28.6)	19 (34.5)	Reference	
Left-sided disease, n (%)	5 (71.4)	24 (43.6)	−11.2 (−29.0 to 5.4)	0.237
Right-sided disease, n (%)	2 (28.6)	31 (56.4)	Reference	
Vascular compression present, n (%)	7 (100.0)	47 (85.5)	−13.0 (−24.4 to 20.1)	0.580
Vascular compression absent, n (%)	0 (0.0)	8 (14.5)	Reference	
Comorbidity present, n (%)	3 (42.9)	20 (36.4)	−2.8 (−22.9 to 13.0)	1.000
No comorbidity, n (%)	4 (57.1)	35 (63.6)	Reference	
Age, median (IQR)	50 (38–61)	54 (40–62)	—	0.559
Symptom duration, median (IQR), years	5 (3–13)	6 (3–10)	—	0.858
Previous interventional procedure before index MVD, n (%)	5 (71.4)	17 (30.9)	−17.7 (−38.8 to −0.7)	0.086
No documented previous interventional procedure before index MVD, n (%)	2 (28.6)	38 (69.1)	Reference	

Categorical variables were compared using two-sided Fisher’s exact test, and continuous variables were compared using the Mann–Whitney U test. Absolute differences represent differences in the proportion of patients achieving complete pain relief between the listed category and the corresponding reference category and are presented as percentage points with 95% confidence intervals. Postoperative BNI and VAS outcomes were available for all 62 patients. Paired CRP measurements were available for 59 patients and paired NLR measurements for 61 patients.

## Data Availability

The data supporting the findings of this study are not publicly available due to institutional and ethical restrictions but are available from the corresponding author upon reasonable request and with permission from the relevant institution.

## Abbreviations

The following abbreviations are used in this manuscript:

MVD	Microvascular decompression
BNI	Barrow Neurological Institute
VAS	Visual analog scale
CRP	C-reactive protein
NLR	Neutrophil-to-lymphocyte ratio
IQR	Interquartile range
SD	Standard deviation
